# Novel plasma protein biomarkers from critically ill sepsis patients

**DOI:** 10.1186/s12014-022-09389-3

**Published:** 2022-12-27

**Authors:** Logan R. Van Nynatten, Marat Slessarev, Claudio M. Martin, Aleks Leligdowicz, Michael R. Miller, Maitray A. Patel, Mark Daley, Eric K. Patterson, Gediminas Cepinskas, Douglas D. Fraser

**Affiliations:** 1 Medicine, Western University, London, ON Canada; 2 Lawson Health Research Institute, London, ON Canada; 3 Pediatrics, Western University, London, ON Canada; 4 Computer Science, Western University, London, ON N6A 3K7 Canada; 5 The Vector Institute for Artificial Intelligence, Toronto, ON M5G 1M1 Canada; 6 Medical Biophysics, Western University, London, ON N6A 3K7 Canada; 7 Clinical Neurological Sciences, Western University, London, ON Canada; 8 Physiology and Pharmacology, Western University, London, ON Canada; 9 London Health Sciences Centre, Room C2-C82, 800 Commissioners Road East, London, ON N6A 5W9 Canada

**Keywords:** Sepsis, Critical care, ICU, Proteomics, Biomarkers

## Abstract

**Background:**

Despite the high morbidity and mortality associated with sepsis, the relationship between the plasma proteome and clinical outcome is poorly understood. In this study, we used targeted plasma proteomics to identify novel biomarkers of sepsis in critically ill patients.

**Methods:**

Blood was obtained from 15 critically ill patients with suspected/confirmed sepsis (Sepsis-3.0 criteria) on intensive care unit (ICU) Day-1 and Day-3, as well as age- and sex-matched 15 healthy control subjects. A total of 1161 plasma proteins were measured with proximal extension assays. Promising sepsis biomarkers were narrowed with machine learning and then correlated with relevant clinical and laboratory variables.

**Results:**

The median age for critically ill sepsis patients was 56 (IQR 51–61) years. The median MODS and SOFA values were 7 (IQR 5.0–8.0) and 7 (IQR 5.0–9.0) on ICU Day-1, and 4 (IQR 3.5–7.0) and 6 (IQR 3.5–7.0) on ICU Day-3, respectively. Targeted proteomics, together with feature selection, identified the leading proteins that distinguished sepsis patients from healthy control subjects with ≥ 90% classification accuracy; 25 proteins on ICU Day-1 and 26 proteins on ICU Day-3 (6 proteins overlapped both ICU days; PRTN3, UPAR, GDF8, NTRK3, WFDC2 and CXCL13). Only 7 of the leading proteins changed significantly between ICU Day-1 and Day-3 (IL10, CCL23, TGFα1, ST2, VSIG4, CNTN5, and ITGAV; P < 0.01). Significant correlations were observed between a variety of patient clinical/laboratory variables and the expression of 15 proteins on ICU Day-1 and 14 proteins on ICU Day-3 (P < 0.05).

**Conclusions:**

Targeted proteomics with feature selection identified proteins altered in critically ill sepsis patients relative to healthy control subjects. Correlations between protein expression and clinical/laboratory variables were identified, each providing pathophysiological insight. Our exploratory data provide a rationale for further hypothesis-driven sepsis research.

**Supplementary Information:**

The online version contains supplementary material available at 10.1186/s12014-022-09389-3.

## Background

Sepsis refers to life-threatening organ dysfunction caused by a dysregulated host response to infection [1, 2]. Septic shock is sepsis with compounded circulatory, cellular, and metabolic dysfunction associated with a higher risk of mortality. Sepsis is a predominant contributor to hospital admissions, as well as patient morbidity and mortality, representing as many as 50% of adult intensive care unit (ICU) admissions and 48% of ICU deaths [3].

Sepsis is manifested by complex immunologic, neuronal, autonomic, hormonal, metabolic and coagulation derangements. These are further influenced by host genetic predisposition and heterogeneity, as well as social determinants of health [4–9]. These dynamic interactions demonstrate the diversity in critical illness pathophysiology, exemplifying the need for investigations rooted in high-throughput technologies, to elucidate their complexity. To this end, we have recently used targeted proteomics to identify novel plasma biomarkers associated with disease outcome in critically ill COVID-19 patients; six novel proteins were identified that predicted ICU mortality with 100% accuracy [10]. Targeted proteomics is valuable for not only identifying proteomic associations with disease progression and outcome, but also for improved understanding of pathophysiology related to specific patient features.

Over the last decade, proteomic analysis of the blood from critically ill patients has provided novel insight into the molecular basis of sepsis [11]. One of the first studies identified inflammatory and acute phase response proteins upregulated in sepsis [11]. Others identified novel biomarkers of lipid transport and coagulation proteins [12], followed by a host of mass spectroscopy and immunologic assays that increased our knowledge of the sepsis proteomes [13–17]. Despite numerous sepsis investigations, biomarkers for disease surveillance and organ dysfunction remain elusive. Moreover, temporal investigations to better understand disease evolution, as well as the host response to therapies, are lacking.

In this study, we performed targeted proteomics with proximity extension assays (PEA) as previously described [18, 19]. The PEA approach relies on antibodies conjugated to complimentary oligonucleotides, which upon binding to a target protein are in close proximity for hybridization and deoxyribonucleic acid (DNA) polymerase-based extension. The complex can then be amplified using microfluidic quantitative polymerase chain reaction (qPCR). PEA offers unparalleled sensitivity and specificity, with measurement of 1161 distinct human plasma proteins in this study.

Our specific study objectives were to: [1] measure plasma protein expression between critically ill sepsis patients and age-/sex-matched healthy control subjects; [2] determine the leading proteins with greatest classification accuracies between cohorts; [3] investigate how these protein expression levels vary temporally in critically ill sepsis patients; and [4] determine correlations between these protein expression levels and relevant clinical/laboratory variables.

## Methods

This study was reviewed and approved by the Western University, Human Research Ethics Board (HREB #6970, “Repository of biological specimens from patients with critical illness and/or traumatic conditions”, March 17, 2021; #17908E, “Analysis of inflammatory markers in blood samples from patients with serious illness”, March 19, 2021). A total 15 patients with sepsis based on *Sepsis-3* criteria [2] were enrolled. Patient characteristics included age, sex, comorbidities, medications, hematologic labs, creatinine, PaO_2_/FiO_2_ (P/F) ratios (arterial to inspired oxygen), chest x-ray findings, Multiple Organ Dysfunction Scores (MODS), Sequential Organ Failure Assessment scores (SOFA), use of antibiotics, anti-virals, corticosteroids, vasopressors, thromboembolism prophylaxis, anti-coagulation status, renal replacement therapy, invasive and non-invasive ventilation, intubation duration and ICU duration. Survival until Day-3 was not an inclusion criterion. For comparison with sepsis patients, 15 healthy control subjects without disease or acute illness were identified from the Translational Research Centre, London, Ontario (HREB #6963, “Repository of control biological specimens from healthy volunteers”, March 22, 2021; Directed by Dr. D.D Fraser; https://translationalresearchcentre.com/) [20, 21]. This study was performed in accordance with the ethical standards of the responsible committee on human experimentation and with the Helsinki Declaration of 1975.

### Blood draws

Standard phlebotomy procedures were used to collect blood. Samples were obtained from critically ill ICU patients via indwelling venous catheters and immediately placed on ice. Maximum volumes were not exceeded per standard phlebotomy protocols. Following collection, samples were transferred to a negative pressure hood; after centrifugation, isolated plasma was aliquoted in 250 µL increments and frozen at − 80 °C. All samples remained frozen until use to avoid repeated freeze–thaw cycles.

### Proximity extension assay

Plasma was thawed for PEA testing (Olink Proteomics, Sweden) as previously described [18, 19]. Specifically, we measured a total of 1161 plasma proteins in the plasma of critically ill sepsis patients and age-/sex-matched healthy controls. Each protein was targeted with two antibodies, labelled with one oligonucleotide each, and having a region complementary to each other. The PEA was performed in three steps: [1] antibody pairs, labelled with unique DNA oligonucleotides, were attached to their target antigen in plasma; [2] oligonucleotides that were brought into proximity hybridized and were extended by a DNA polymerase; and [3] the newly formed DNA barcode was amplified using qPCR. Individual samples were screened based on quality controls for immunoassay and detection, as well as degree of hemolysis. Intra-assay variability was minimized via robotic pipetting for volume accuracy, and through normalization using three specifically engineered internal controls that were added to each sample, including one control for the incubation, one for the extension and one for the amplification. External negative control and plate control samples were included in each sample plate in triplicate to improve inter-assay precision. Following proteomic quality control, all 30 (15 healthy control subjects and 15 critically ill sepsis) participants were deemed suitable for analysis. The data generated were expressed as relative quantification on the log2 scale of normalized protein expression (NPX) values. NPX values were rank‐based normal transformed for further analyses.

### Population statistics

Medians (interquartile ranges [IQRs]) and frequency (%) were used to report ICU patient baseline characteristics for continuous and categorical variables, respectively. Group differences in baseline characteristics between sepsis patients on Day-1 and Day-3 were examined with Wilcoxon signed-rank tests given the dependency of the data (GraphPad Prism Version 8.4.0; San Diego, California USA). Statistical differences in protein expression between healthy controls and sepsis patients on either Day-1 or Day-3 were examined with Mann–Whitney U tests. Temporal differences in protein expression amongst sepsis patients were analyzed using Wilcoxon signed-rank tests. Statistical significance for protein expression between critically ill sepsis patients and healthy control subjects was adjusted for multiple comparisons using the Bonferroni correction (P < 0.05 post correction). As fewer proteins were analyzed for temporal differences between ICU Day-1 and Day-3, a P < 0.01 was considered statistically significant. Heat maps depicting Pearson correlation values between proteins and clinical or biochemical parameters were created in R (http://www.r-project.org) using the ggplot2 version 3.3.3 package. Significant correlations had a Pearson R-value of either 0.5 to 1.0 or -0.5 to -1.0 and a P < 0.05. Pearson correlation values assessing dynamic change (ICU Day-3 values minus ICU Day-1 values) between proteins and clinical or biochemical parameters were examined using SPSS v.28 (IBM Corp., Armonk, NY, USA). There were no missing data for any clinical outcomes.

### Machine learning

Machine learning was implemented to identify protein variation underlying differences in the proteomes of ICU Day-1 and Day-3 sepsis patients, versus healthy controls. Analyte data were visualized with a nonlinear dimensionality reduction on the full matrix using the t-distributed stochastic nearest neighbour embedding (t-SNE) algorithm we have previously characterized [22]. t-SNE assumes that the ‘optimal’ representation of the data lies on a manifold with complex geometry, but low dimension, embedded in the full dimensional space of the raw data [23]. For feature selection, the raw data for each subject were ingested across subjects. A random forest classifier was trained on the variables to predict sepsis status. A random forest is a set of decision trees and, consequently, we were able to interrogate this collection of trees to identify the features that had the highest predictive value, or those features that frequently appeared near the top of the decision tree, for determining sepsis patients versus healthy controls. The feature matrices for ICU Day-1 and Day-3 critically ill sepsis patients were classified for patient outcome using a three-fold cross validation with a random forest of one hundred trees and max depth of six trees to reduce overfitting [24]. A Boruta feature selection method, based on random forest classifiers, was used to develop a reduced model using a training set (70%) and verified with a testing set (30%). The hyperparameter for the Boruta algorithm “percentile” was set to 90. All machine learning analysis was done using Python 3.9, Scikit-Learn (v. 24.0) and Boruta Py (v. 0.3) [25, 26].

## Results

Demographic, clinical and laboratory parameters for the 15 critically ill sepsis patients, as defined by *Sepsis-3* criteria [2], are presented in Table 1. A pathogen was identified in one third of patients and consisted of Methicillin-Resistant *Staphylococcus aureus* (sputum), Methicillin-Susceptible *Staphylococcus aureus* (blood), *Streptococcus pneumoniae* (sputum), *Metapneumovirus* (sputum) or *Micrococcus species* (blood). All patients were intubated and mechanically ventilated at some point throughout their ICU admission (median 4 days; IQR 3–4), and three quarters required vasoactive medications. The median ICU stay was 5 days (IQR 4–6). Only 1 of the 15 sepsis patients died, occurring in the ICU on Day-3 after blood work was drawn and clinical variables collected.

**Table 1:** Demographic and clinical data

Variables	Healthy control subjects	Critically ill sepsis patients ICU day-1	Critically ill sepsis patients ICU day-3	P-value
n	15	15	15	1.000
Age in years, median (IQR)	55 (51–61)	56 (51–61)	56 (51–61)	0.448
Sex, female: male	9:6	9:6	9:6	1.000
MODS, median (IQR)	–	7 (5.0–8.0)	4 (3.5–7.0)	0.055
SOFA, median (IQR)	–	7 (5.0–9.0)	6 (3.5–7.0)	0.012*
Laboratory, median (IQR)				
Hemoglobin (g/L)	–	128.0 (109.0–147.0)	110.0 (87.0–120.0)	< 0.001*
White Blood Cell (10^9^/L)	–	15.9 (11.5–22.7)	12.2 (7.3–14.3)	0.002*
Neutrophils (10^9^/L)	–	12.9 (8.6–15.7)	9.8 (5.4–11.9)	0.006*
Lymphocytes (10^9^/L)	–	1.2 (0.7–1.7)	1.2 (0.7–1.6)	0.528
Platelets (10^9^/L)	–	182.0 (107.0–259.0)	116.5 (79.0–189.0)	< 0.001*
Partial thromboplastin time (sec)	–	23.0 (21.0–29.0)	24.0 (22.0–27.0)	0.805
International normalized ratio ([PTt/PTn]ISI)	–	1.0 (1.1–1.2)	1.0 (1.0–1.2)	0.313
Creatinine (mmol/L)	–	79.0 (56.0–106.0)	54.5 (47.0–70.0)	0.065
Lactate (mmol/L)	–	1.7 (0.9–3.2)	1.0 (0.9–1.2)	0.021*
Chest radiograph, n (%)				
Bilateral pneumonia	–	3 (20.0)	3 (20.0)	1.000
Unilateral pneumonia	–	7 (46.7)	7 (46.7)	1.000
Interstitial infiltrates	–	1 (6.7)	1 (6.7)	1.000
Normal	–	4 (26.7)	4 (26.7)	1.000
P/F Ratio, median (IQR)	–	164.0 (130.0–273.0)	188.0 (151.5–282.0)	0.321
Interventions, n (%)				
Antibiotics	–	15 (100.0)	15 (100.0)	1.000
Antivirals	–	1 (6.7)	0 (0)	0.309
Steroids	–	4 (26.7)	3 (20.0)	0.667
Vasoactive medications	–	11 (73.3)	5 (33.3)	0.028*
High Flow Nasal Cannula	–	0 (0)	1 (6.7)	0.309
Noninvasive MV	–	2 (13.3)	0 (0)	0.143
Invasive MV	–	15 (100.0)	12 (80.0)	0.068
Sepsis diagnosis, n (%)				
Suspected	–	10 (66.7)	–	–
Confirmed	–	5 (33.3)	–	–
Comorbidities, n (%)				
Hypertension	–	9 (60.0)	–	–
Diabetes	–	5 (33.3)	–	–
Chronic Kidney Disease	–	1 (6.7)	–	–
Coronary Artery Disease	–	0 (0)	–	–
Chronic Heart Failure	–	1 (6.7)	–	–
Cancer	–	1 (6.7)	–	–
COPD	–	3 (20.0)	–	–
Baseline medications, n (%)				
Antiplatelet	–	6 (40.0)	–	–
Anticoagulants	–	3 (20.0)	–	–

^*^P < 0.005

When comparing ICU patient clinical data on Day-1 versus Day-3, statistically significant decreases in white blood cell count, neutrophils, lactate and the use of vasoactive medications were observed (P < 0.05), suggesting overall patient improvement. Sepsis patients were intubated for a median of 4 days (IQR 3–4), and the median length of hospital admission was 12 days (IQR 7–16).

**Fig. 1:**
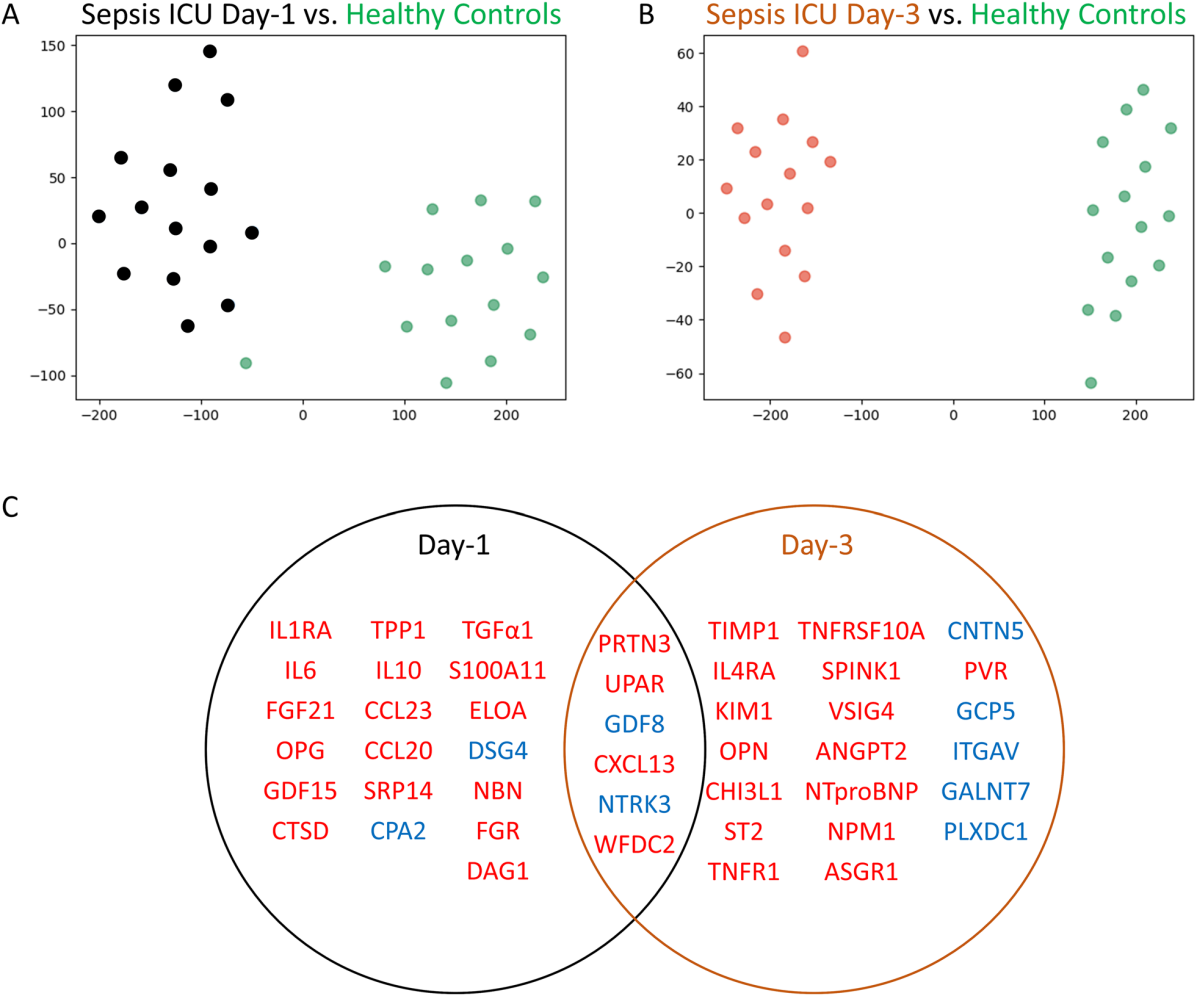
Targeted proteomics accurately differentiates critically ill sepsis patients from healthy controls. In the upper section, t-SNE plots depict the separation between sepsis patients and healthy controls. Age- and sex-matched subjects plotted in 2D following dimensionality reduction of their respective proteomes by t-distributed stochastic neighbour embedding. Axes are dimensionless. The dimensionality reduction shows that based on plasma proteome, the two cohorts are distinct and easily separable. **A** Black dots represent ICU Day-1 patients and green dots represent healthy controls. **B** Orange dots represent ICU Day-3 patients and green dots represent healthy controls. **C** Proteins that feature selection identified as classifying proteins on both ICU Day-1 and Day-3 are shown in the Venn-diagram with overlap. Proteins in Red text were upregulated when compared to healthy controls, whereas proteins in Blue text were downregulated when compared to healthy controls

Of the 1161 plasma proteins measured using PEA, feature selection identified those with a 90% or greater classification accuracy for identifying a critically ill sepsis patient when compared to healthy control subjects. Using the 90% classification model, 25 leading proteins were identified on ICU Day-1 (Table 2) and 26 leading proteins on ICU Day-3 (Table 3). Respective t-SNE plots of the leading classifying proteins illustrate that the proteome of critically ill sepsis patients was distinct, recognizable and easily separated from age- and sex-matched healthy control subjects (Fig. 1A, B). A total of 6 leading proteins overlapped on ICU Day-1 and Day-3 (PRTN3, UPAR, GDF8, CXCL13, NTRK3 and WFDC2; Fig. 1C).

**Table 2:** Median plasma expression of the top 25 classifying proteins, ICU Day-1

Protein	Healthy controls	Sepsis day-1	Change	P-value	Corrected P-value
IL1RA	15.38 (13.22–19.66)	109.61 (83.37–142.19)	↑	< 0.0001	0.0015
IL6	4.16 (3.33–4.66)	169.24 (103.69–249.87)	↑	< 0.0001	0.0015
FGF21	58.31 (30.80–71.63)	498.68 (196.18–1735.47)	↑	< 0.0001	0.0015
OPG	2.95 (2.51–3.27)	7.47 (5.29–10.82)	↑	< 0.0001	0.0015
GDF15	3.90 (3.35–4.51)	15.80 (12.49–30.66)	↑	< 0.0001	0.0015
PRTN3	6.04 (4.79–7.27)	22.72 (17.19–29.89)	↑	< 0.0001	0.0015
UPAR	6.56 (5.83–7.88)	15.45 (11.52–30.35)	↑	< 0.0001	0.0015
CTSD	3.17 (2.67–3.42)	6.75 (4.54–8.26)	↑	< 0.0001	0.0015
DAG1	0.94 (0.88–1.01)	1.34 (1.25–1.51)	↑	< 0.0001	0.0015
TPP1	19.20 (17.12–20.23)	32.29 (29.31–50.82)	↑	< 0.0001	0.0015
IL10	8.31 (7.20–10.21)	55.64 (32.67–98.75)	↑	< 0.0001	0.0015
CCL23	628.15 (434.83–729.69)	2299.48 (1512.89–3088.85)	↑	< 0.0001	0.0015
CCL20	83.66 (62.37–101.75)	570.22 (401.45–819.50)	↑	< 0.0001	0.0015
CPA2	830.36 (591.96–906.17)	162.32 (79.99–329.63)	↓	< 0.0001	0.0015
GDF8	11.32 (9.56–16.78)	2.84 (2.54–3.48)	↓	< 0.0001	0.0015
NTRK3	107.56 (103.68–118.03)	67.96 (54.14–79.73)	↓	< 0.0001	0.0015
TGFα1	4.21 (4.04–5.21)	9.86 (7.20–16.83)	↑	< 0.0001	0.0015
WFDC2	374.19 (323.03–402.35)	851.42 (706.71–1441.42)	↑	< 0.0001	0.0015
CXCL13	134.98 (116.85–164.03)	408.14 (300.90–493.60)	↑	< 0.0001	0.0015
FGR	2.85 (2.20–3.29)	10.66 (8.25–15.97)	↑	< 0.0001	0.0031
S100A11	10.24 (9.33–11.50)	20.31 (16.91–22.85)	↑	< 0.0001	0.0031
DSG4	5.77 (5.55–8.46)	2.80 (2.04–3.83)	↓	< 0.0001	0.0046
ELOA	0.47 (0.46–0.57)	1.02 (0.86–1.44)	↑	< 0.0001	0.2090
NBN	1.82 (1.59–2.56)	4.89 (4.04–6.99)	↑	< 0.0001	0.2090
SRP14	2.87 (2.43–3.63)	9.93 (7.00–19.83)	↑	< 0.0001	0.2090

**Table 3:** Median plasma concentration of the top 26 classifying proteins, ICU Day-3

Protein	Healthy Controls	Sepsis Day-3	Change	P-Value	Corrected P-Value
TIMP1	17.58 (16.19–17.89)	40.17 (27.01–54.83)	↑	< 0.0001	0.0016
IL4RA	2.08 (1.90–2.22)	6.56 (3.63–8.15)	↑	< 0.0001	0.0016
PRTN3	6.04 (4.79–7.27)	21.62 (15.90–27.30)	↑	< 0.0001	0.0016
UPAR	6.56 (5.83–7.88)	13.75 (11.00–27.40)	↑	< 0.0001	0.0016
OPN	11.85 (9.44–15.29)	70.09 (43.82–78.26)	↑	< 0.0001	0.0016
CHI3L1	6.60 (5.82–8.32)	51.95 (27.92–68.54)	↑	< 0.0001	0.0016
ST2	5.00 (4.48–5.47)	44.50 (16.73–72.03)	↑	< 0.0001	0.0016
TNFRSF10A	5.33 (4.82–5.92)	10.20 (8.48–14.02)	↑	< 0.0001	0.0016
VSIG4	17.45 (14.62–19.43)	94.61 (47.58–203.81)	↑	< 0.0001	0.0016
ANGPT2	3.94 (3.37–4.07)	9.90 (7.78–16.06)	↑	< 0.0001	0.0016
NTproBNP	7.94 (5.64–12.64)	204.13 (66.21–817.22)	↑	< 0.0001	0.0016
GDF8	11.32 (9.56–16.78)	2.11 (1.61–2.88)	↓	< 0.0001	0.0016
PVR	218.29 (199.04–243.63)	309.93 (285.85–389.24)	↑	< 0.0001	0.0016
GCP5	14.86 (12.61–16.55)	6.36 (4.92–8.15)	↓	< 0.0001	0.0016
NTRK3	107.56 (103.68–118.03)	62.54 (49.32–70.67)	↓	< 0.0001	0.0016
ITGAV	26.03 (24.62–28.56)	15.99 (11.29–17.31)	↓	< 0.0001	0.0016
WFDC2	374.19 (323.03–402.35)	985.66 (767.97–1208.01)	↑	< 0.0001	0.0016
CXCL13	134.98 (116.85–164.03)	446.75 (279.79–716.64)	↑	< 0.0001	0.0016
GALNT7	16.62 (14.71–18.76)	9.52 (7.20–10.56)	↓	< 0.0001	0.0016
KIM1	110.58 (93.33–161.57)	590.88 (461.90–854.33)	↑	< 0.0001	0.0032
TNFR1	19.92 (17.61–23.92)	49.37 (33.71–86.61)	↑	< 0.0001	0.0032
SPINK1	7.53 (6.75–9.08)	75.56 (22.32–239.37)	↑	< 0.0001	0.0032
ASGR1	10.53 (9.60–11.96)	32.71 (20.44–42.41)	↑	< 0.0001	0.0032
CNTN5	24.25 (21.81–25.63)	8.72 (6.28–10.84)	↓	< 0.0001	0.0032
PLXDC1	7.34 (6.79–8.14)	4.71 (3.93–5.21)	↓	< 0.0001	0.2174
NPM1	11.89 (8.82–13.24)	30.37 (22.76–38.50)	↑	< 0.0001	0.7839

The median plasma protein expression of the classifying proteins in both healthy control subjects and critically ill sepsis patients are shown for ICU Day-1 (Table 2) and Day-3 (Table 3), with their respective Bonferroni corrections. An alphabetized list of these proteins with brief descriptions are provided in Additional file 1: Table S1. While the majority of normalized plasma protein expression were increased in critically ill sepsis patients compared to healthy control subjects, a small number were decreased. For the latter, CPA2, GDF8, NTRK3 and DSG4 all had lower median plasma expression on ICU Day-1 when compared to healthy control subjects (Table 2). Similarly, on ICU Day-3, CNTN5, GDF8, GCP5, NTRK3, ITGAV, GALNT7 and PLXDC1 had lower median plasma expression as compared to healthy controls (Table 3).

**Fig. 2:**
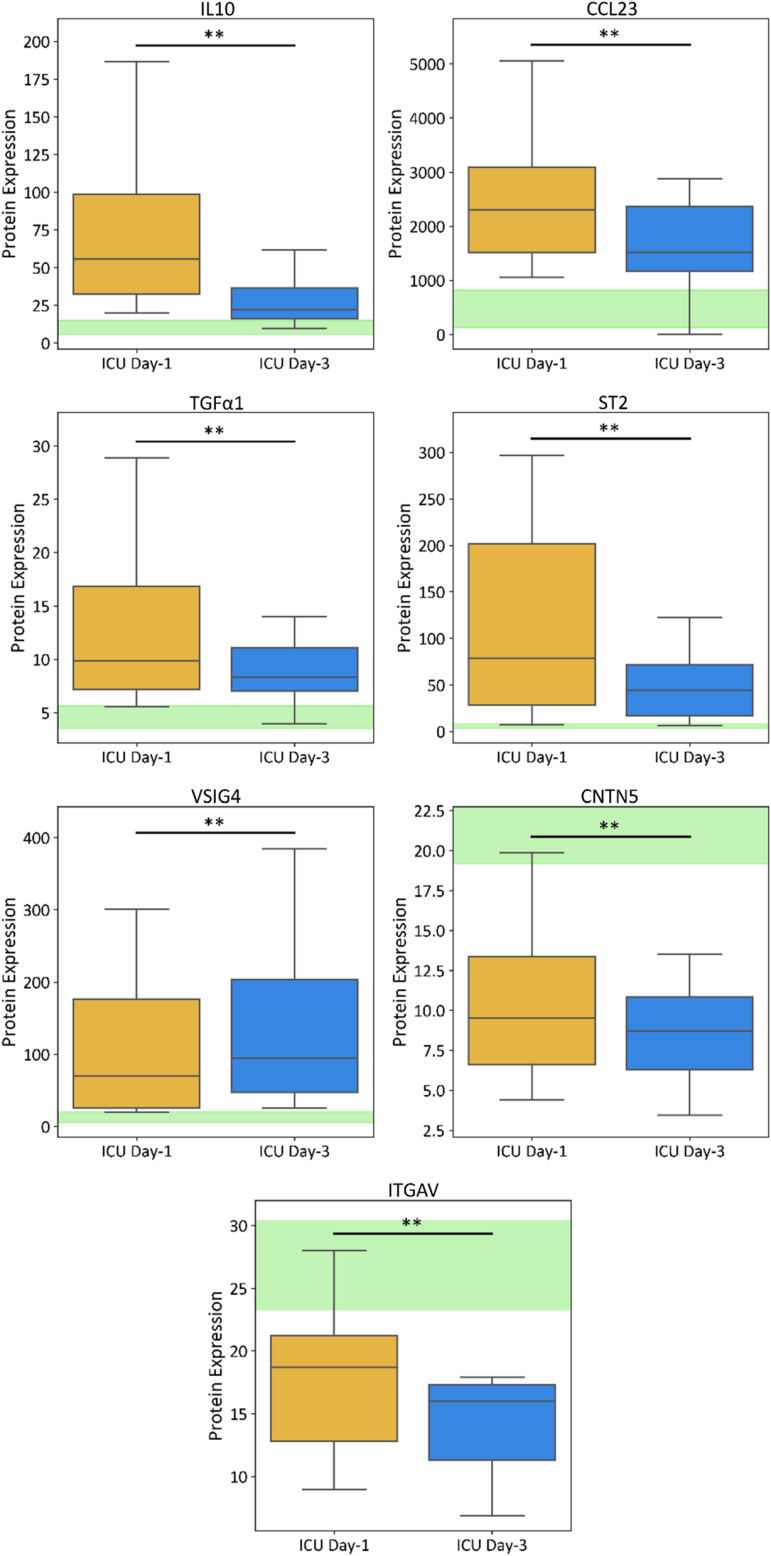
Significant temporal differences in plasma protein expression from sepsis patients. Box plots illustrating statistically significant differences between sepsis patients on ICU Day-1 versus ICU Day-3. The green shading illustrates healthy control protein expression (5–95% percentiles). Three ICU Day-1 classifying proteins significantly decreased in concentration by Day-3 (IL-10, CCL23, and TGFα1), and three ICU Day-3 classifying proteins significantly decreased in concentration by Day-3 (ST2, CNTN5, and ITGAV). Only one ICU Day-3 classifying protein was upregulated by ICU Day-3 (VSIG4). Median differences in protein concentrations were analyzed using Wilcoxon signed-rank tests, with P < 0.01 deemed statistically significant

Only a small number of plasma proteins significantly changed in critically ill sepsis patients from ICU Day-1 to Day-3**.** Among the ICU Day-1 classifying proteins IL-10, CCL23, and TGFα1, significantly decreased in sepsis patient plasma by Day-3 (Fig. 2; Additional file 1: Table S2). Of the ICU Day-3 classifying proteins, ST2, CNTN5, and ITGAV decreased significantly in median level from ICU Day-1 to Day-3 and VSIG4 increased significantly in sepsis patient plasma between Day-1 and Day-3 (Fig. 2B).

**Fig. 3:**
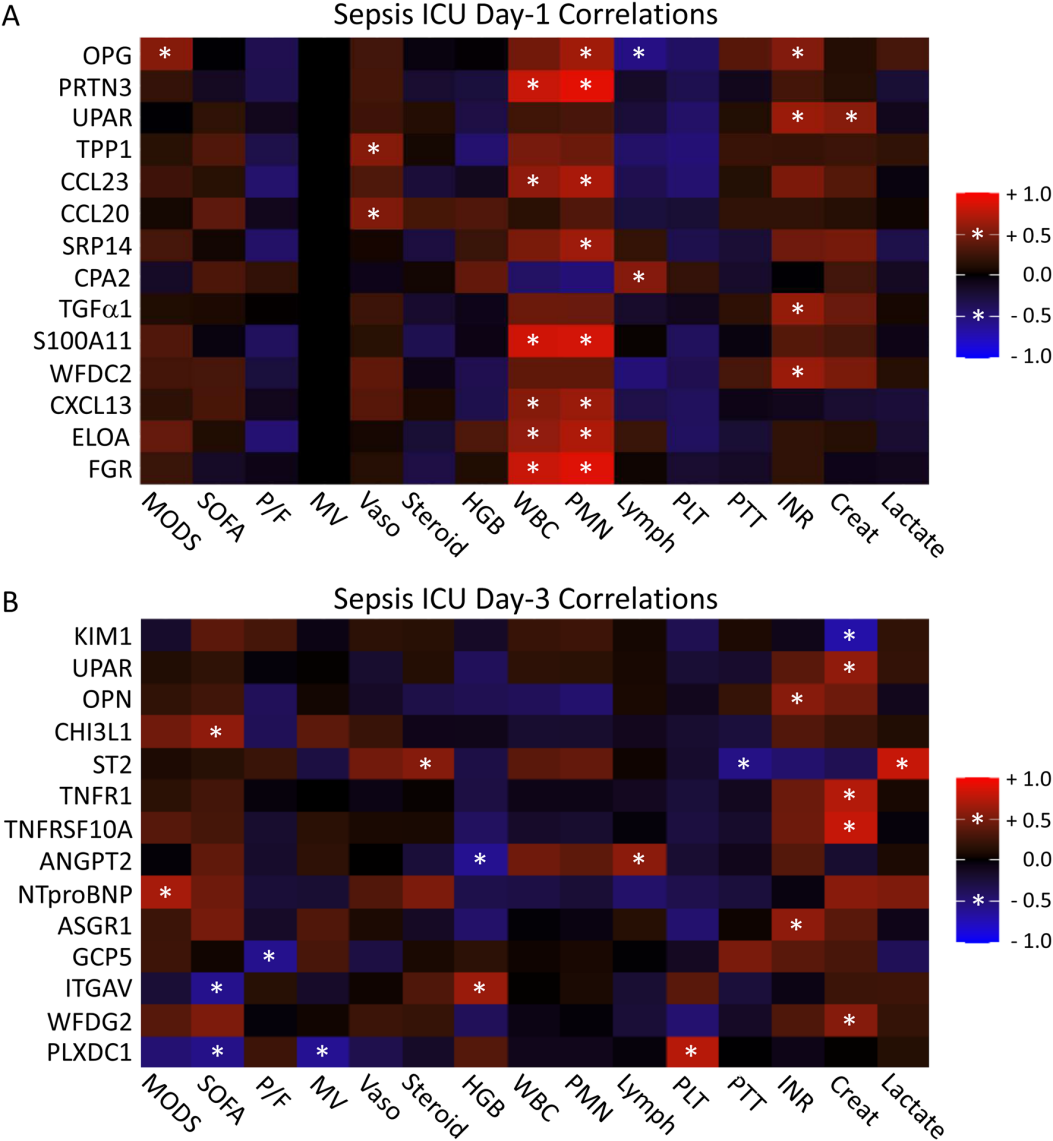
Correlations between normalized plasma protein expression and sepsis patient parameters on ICU Day-1 and Day-3. Heat maps of rank-based classifying proteins reported in Fig. 1 on ICU Day-1 (**A**) and ICU Day-3 (**B**) are illustrated (y -axis) along with patient parameters (x-axis). Only proteins that showed a significant correlation (P < 0.05) with at least one biochemical or clinical parameter are illustrated. Significant correlations had a Pearson R-value of ≥ 0.5 or ≤ -0.5 and P < 0.05, denoted by *. Positive correlations are depicted in red and negative correlations in blue. *MODS* Multiple Organ Dysfunction Score, *SOFA* Sequential Organ Failure Assessment, *P/F* arterial partial pressure of oxygen divided by the fraction of inspired oxygen concentration, *MV* mechanical ventilation, Vaso vasopressors, *HgB* hemoglobin concentration, *WBC* white blood cell count, *PMN* neutrophil count; *Lymph* lymphocyte count, *PLT* platelet count, *PTT* partial thromboplastin time, *INR* international normalized ratio, *Creat* creatinine concentration

We then investigated whether the normalized protein expression in critically ill sepsis patients were correlated with their clinical, hematologic and biochemical parameters. On ICU Day-1 (Fig. 3A), OPG positively correlated with increasing MODS. TPP1 and CCL20 positively correlated with the use of vasopressors. In terms of hematologic parameters, PRTN3, CCL23, S100A11, CXCL13, ELOA, and FGR all positively correlated with increased white blood cell and neutrophil counts. OPG and SRP14 also positively correlated with neutrophilia. CPA2 positively correlated with lymphocyte count, and OPG negatively correlated with lymphocyte count. In terms of biochemical parameters, OPG, UPAR, TGFα1 and WFDC2 positively correlated with increasing international normalized ratio (INR). UPAR positively correlated with creatinine. Correlations of dynamic change (Day 3 minus Day 1) between normalized plasma protein expression and sepsis patient parameters are provided in Additional file 1: Table S3. In this latter analyses, positive correlations reflect movement of both clinical parameter and protein in the same direction, either increased or decreased, whereas negative correlations indicate divergence of the clinical parameter and protein in opposite directions.

Significant correlations between classifying proteins and patient variables on ICU Day-3 are shown in Fig. 3B. NTproBNP positively correlated with MODS. CHI3L1 positively correlated, while ITGAV and PLXDC1 negatively correlated, with SOFA score. GCP5 negatively correlated with P/F ratio, and PLXDC1 negatively correlated with mechanical ventilation. ST2 positively correlated with stress-steroid administration. ANGPT2 negatively correlated, and ITGAV positively correlated, with hemoglobin. ANGPT2 also positively correlated with lymphocyte count. PLXDC1 positively correlated with platelets. OPN and ASGR1 positively correlated with INR. UPAR, TNFR1, TNFRSF10A and WFDC2 positively correlated, while KIM1 negatively correlated, with creatinine. ST2 negatively correlated with partial thromboplastin time, while positively correlated with lactate.

## Discussion

In this study, we performed targeted, high-throughput plasma proteomic profiling of critically ill sepsis patients on ICU Day-1 and Day-3. We used PEA [18, 19] to measure 1161 plasma proteins, and confirmed distinct proteomes in sepsis patients. Feature selection identified proteins that differentiate between critically ill sepsis patients and healthy control subjects with greater than 90% accuracy, and provided a rank order of importance based on their ability to differentiate cohorts. Moreover, we identified correlations between normalized protein expression and clinical, hematological and biochemical parameters. These data will be valuable for future hypothesis-driven studies [22], particularly those investigating disease surveillance, organ dysfunction and outcome prediction. In addition, the proteins identified here can be further evaluated with bioinformatics to aid our understanding of signalling pathways and potential therapeutics targets.

The baseline characteristics and comorbidities recorded in our critically ill sepsis patients were similar to other studies that profiled plasma proteomes using a variety of immunoassay methods [10, 22]. Pathogens were identified in 33% of patients (4 bacteria and 1 viral). All sepsis patients required mechanical ventilation, with most patients showing acute lung injury. Vasoactive medications were required in 73% of sepsis patients. Despite relatively high MODS and SOFA scores, our sepsis cohort had a higher survival rate (14 out of 15 patients) than expected based on previous literature [27].

We identified novel sepsis protein biomarkers using PEA and feature selection. A threshold of 90% classification accuracy was used to narrow the number of clinically relevant proteins for correlation studies: 25 proteins on ICU Day-1 and 26 proteins on ICU Day-3. The following 6 proteins overlapped on ICU Day-1 and Day-3: PRTN3, UPAR, GDF8, NTRK3, WFDC2 and CXCL13.

Only a small number of plasma proteins changed significantly between ICU Day-1 and Day-3 in critically ill sepsis patients, with decreased expression in IL10, CCL23, TGFα1, ST2, CNTN5, and ITGAV, and increased expression of VSIG4. CCL23 is a chemokine that is highly chemotactic for T-cells and monocytes, and has reported age-related differences in sepsis [28]. IL10 is an anti-inflammatory cytokine previously reported to be upregulated in septic shock, whose impact on clinical outcome is still unclear [29, 30]. TGFα1 is a mitogenic peptide (signalling through epidermal growth factor receptors triggering a kinase cascade) that has been found in bronchoalveolar lavage samples in ARDS correlating with lung injury and mortality [31]. ST2 is a member of the IL1R family that has roles in cardiac disease and cardio protection, and serves prognostic value after myocardial infarction. CNTN5, a member of the immunoglobulin superfamily, is a glycosylphosphatidylinositol-anchored membrane protein known to mediate nervous system development, which is relatively unexplored in sepsis [32, 33]. ITGAV is an integrin mediating cell adhesion (discussed below). VSIG4 encodes immunoglobulin domains that are structurally similar to the B7 family proteins, acting as a negative regulator of T-cell responses [34]. Taken together, each of the above proteins help to coordinate inflammatory responses. Both IL10 and TGF family proteins have been previously investigated as therapeutic targets in interventional trials, largely due to their measured serum changes in sepsis patients [35]. Polymorphisms in IL10 genes have also been associated with altered sepsis mortality and risk of ICU admission, highlighting IL10 as a potential therapeutic target [36]. Given that little is known about temporal protein expression in sepsis patients receiving ICU therapies [37, 38], these identified proteins may represent a targeted pool that hold specific value to understanding the host responses. Temporal proteomic analysis may also identify specific proteins with unique roles in disease pathophysiology beyond the acute illness phase, and provide prognostic information for those patients who have longer ICU admissions [39].

Significant correlations occurred between several proteins and parameters of disease severity, ventilation, and need for vasoactive agents. On ICU Day-1, OPG uniquely correlated with MODS, consistent with previous studies showing OPG as a biomarker of disease severity and mortality in sepsis [40]. OPG was first implicated in osteoclast regulation, yet recent evidence suggests emerging roles in vascular and immune biology [41]. Recently, OPG levels in sepsis were shown to correlate with markers of endothelial dysfunction, raising the possibility that endothelial dysfunction is mediated by OPG and resulting in end-organ dysfunction [42]. By ICU Day-3, only NTproBNP positively correlated with MODS. NTproBNP is secreted by the cardiac ventricles in response to ventricular expansion and pressure overload to promote natriuresis [43, 44]. With sepsis, NTproBNP expression may be influenced by fluid resuscitation and volume overload, resulting in increased myocardial stretch. Indeed, NTproBNP levels are used clinically for cardiac and perioperative risk stratification [45], suggesting it may provide similar risk stratification in sepsis patients.

Only CHI3L1 positively correlated with SOFA score. CHI3L1 is known to regulate tissue injury, repair and inflammation with strong associations in asthma, cancer, diabetes, cirrhosis, and coronary artery disease [46]. Moreover, previous cohort studies have documented the ability of CHI3L1 to predict outcome in infectious and inflammatory diseases [47, 48], highlighting this protein as an inadequately studied modulator of sepsis [47]. In contrast, ITGAV and PLXDC1 negatively correlated with SOFA score on ICU Day-3. ITGAV is an integral membrane protein, which regulates signal transduction, gene expression, proliferation, apoptosis, angiogenesis, invasion and metastasis [49]. ITGAV is also thought to preserve hematopoietic stem cells in the bone marrow [50], and regulate adipocyte proliferation and differentiation [51]. PLXDC1 is expressed on vascular endothelium, serving as a receptor for PEDF, with involvement in capillary morphogenesis [52, 53]. Collectively, proteins that correlate with MODS and SOFA scores may complement these clinically validated prognostication scores to provide more accurate diagnostic certainty [54].

Correlations were not significant for acute lung injury, including both P/F ratio and rates of mechanical ventilation, on ICU Day-1; however, these correlations were significant by Day-3. GCP5 negatively correlated with P/F ratio. GCP5 is a component of the gamma tubulin complex facilitating microtubule nucleation and centrosome assembly [55], suggesting a role in cell division and migration. The exact clinical significance of GCP5 in lung injury remains unknown. PLXDC1 negatively correlated with mechanical ventilation, as well as SOFA (reported above). As a receptor for PEDF, PLXDC1 may play a protective role in the pulmonary system. For example, PEDF is associated with a variety of pulmonary diseases such as idiopathic pulmonary fibrosis, lung cancer and COPD [56–59]. PEDF also has been associated with capillary permeability and septic shock [60, 61]. Given the role of PLXDC1 endothelial regulation, further investigation in sepsis is warranted with particular focus on alveolar-capillary unit.

Vasoactive agents are often necessary to maintain adequate end-organ perfusion. TPP1 and CCL20 positively correlated with the need for vasopressors on ICU Day-1. The serine protease TPP1 regulates lysosomal proteins [62], as well as mitochondrial trafficking and cellular metabolism [63], suggesting a role in cellular metabolism [64].The chemokine CCL20 regulates innate immunity and inflammation in skin and mucosa [65], is upregulated by LDL in smooth muscle of vascular structures, and is a reported biomarker for aortic aneurysms [66, 67].

Multiple proteins expressed by immune cells positively correlated with either total white blood cell count or neutrophil count on ICU Day-1, including OPG, PRTN3, CCL23, SRP14, S100A11, CXCL13, ELOA, and FGR**.** Uniquely, OPG (described above), positively correlated with neutrophilia and negatively correlated with lymphocytosis. The serine protease PRTN3 is produced and secreted by granulocytes, and is implicated in endothelial dysfunction during sepsis [68], while also having roles in organizing immune responses and autoimmune disease [69–71]. The chemokines CCL23 and CXCL13 attract immune cells [72], with CXCL13 affecting lymph node organization, B-cell migration, and chronic inflammatory diseases [73, 74]. SRP14, which traffics secretory proteins through the endoplasmic reticulum, and ELOA, a subunit of the transcription factor B (SIII) complex, may aid protein synthesis by immune cells during sepsis [75, 76]. S100A11 and FGR both regulate cellular migration and motility [77–79]. Their positive correlations with leukocyte and neutrophil counts may reflect leukocytes extravasating into tissues during sepsis. CPA2, a carboxypeptidase expressed by pancreatic tissue [80], was positively correlated on ICU Day-1 with lymphocytosis. As there are few published studies on CPA2 and immune regulation, further investigations are warranted. Interestingly, OPG and CPA2, emerge as potential key early regulators of either a neutrophilic or lymphocytic immune response in sepsis. Collectively, the identified correlations highlight the complexity of host response in sepsis, and suggest that these proteins could play central roles in cellular activation and migration.

By ICU Day-3, there were far fewer significant hematologic correlations compared to ICU Day-1. ITGAV (described above) positively correlated with hemoglobin titre. ANGPT2 negatively correlated with hemoglobin titre, but positively correlated with lymphocytosis. ANGPT2 prognosticates shock and death in critically ill patients [81], and is expressed by the vascular endothelium where it interacts with Tie and VEGF proteins to regulate angiogenesis, while demonstrating both pro- and anti-inflammatory properties [82]. PLXDC1 (described above) correlated with platelets on ICU Day-3.

There were significant correlations with INR. On ICU Day-1, OPG, TGFα1, WFDC2 and UPAR positively correlated with INR. OPG (discussed above) has emerging roles in vascular and immunobiology [41]. TGFα1 regulates cell proliferation, differentiation and development through binding epidermal growth factor receptors, with a specific role in tissue regeneration [83, 84]. WFDC2, also known as HE4, is an anti-protease that promotes angiogenesis [85–87] and is associated with kidney [88, 89] and lung [90] fibrosis. Elevated UPAR has been associated with lupus, cardiovascular disease, pancreatitis, cirrhosis and COVID-19 pathogenesis [91]. On ICU Day-3, OPN and ASGR1 positively correlated with INR. OPN is a matricellular protein that interacts with integrins to mediate cell motility, and promotes cell-mediated immune responses; it is involved in atherosclerosis, glomerulonephritis, cancer, and chronic inflammation, while also serving in biomineralization and inhibiting vascular calcification [92]. OPN was reported to be elevated in sepsis patients previously [93]. ASGR1 is a transmembrane protein that mediates endocytosis and lysosomal degradation of glycoproteins [94]. INR correlations suggest that these proteins may contribute to the consumptive coagulopathies and hepatic injury that often occur in sepsis, which greatly impact mortality and morbidity [95, 96].

KIM1, TNFR1, TNFRSF10A, UPAR (described above) and WFDC2 (described above) were correlated with creatinine, suggesting an association of these proteins with renal injury. KIM1, a type-I cell surface glycoprotein, negatively correlated with creatinine. As an acute marker of early acute kidney injury, KIM1 is predictive of long-term renal outcome [97] and is reported to have protective functions in renal ischemia–reperfusion injury, promote renal graft recovery, have a role in renal cancer pathophysiology and serve as a biomarker for sepsis-induced acute kidney injury [98–102]. The tumor necrosis and transforming growth factor protein families have well-known roles in cellular migration, growth, differentiation, and tissue repair [103], as well as critical roles in mediating the cytokine storm in sepsis [104]. As renal failure is an independent risk factor for morality in sepsis, the identification of protein markers associated with renal dysfunction may provide important prognostic information [105, 106].

Lactate is a critical marker of end-organ perfusion in sepsis. ST2 positively correlated with lactate on ICU Day-3. ST2, has been best characterized in cardiac disease, whereby myocardial stretch or myocyte injury results in ST2 upregulation and secretion of soluble ST2 that binds with IL33 to regulate cardiac response to injury [107]. ST2 also has elucidated roles in T-cell regulation, through interplay with IL33 and downstream transcriptional effects [108]. Relevant to sepsis, ST2 correlates with mortality and facilitates endothelial permeability [109, 110]. Moreover, ST2 negatively correlated with PTT and positively correlated with steroid therapy in our study. The latter correlation between ST2 and steroid dosing may relate to hydrocortisone-induced shock reversal [111], and subsequent improvements in lactate. New biomarkers of end-organ perfusion could impact management decisions in sepsis, as lactate can be unreliable in certain circumstances such as administration of vasoactive agents or confounding causes of Type B and D lactic acidosis [112, 113].

Correlations of dynamic change from ICU Day-1 to Day-3 between normalized plasma protein expression and sepsis patient parameters revealed interesting temporal findings, particularly for plasma chemokines. For example, a temporal decrease in CCL23 correlated with increased P/F ratio and decreased lactate, suggesting improved lung function and oxygen delivery. A decrease in CCL20 expression with time also correlated with decreased lactate. In contrast, a temporal increase in CXCL13 expression correlated with increased MODS and both decreased P/F ratio and worsening coagulopathy (increased INR and PTT).

Our study has several limitations. First, due to the cost of proteomics, we studied a relatively small sample size; however, our sample size is on par with a subset of other published exploratory studies investigating proteomic variation in sepsis [114]. Second, the limited dataset could also result in the random forest model overfitting; however, our use of conservative parameters would help mitigate this potential risk (three-fold cross-validation, number of trees was limited to 100 and the maximum depth of the trees was limited to 6). Third, given our incomplete knowledge of sepsis, and the proteins identified here within, we cannot propose direct links or mechanisms. Nonetheless, our exploratory study provides excellent background for hypothesis-generation. Fourth, given the improved SOFA scores, lactates and vasopressor requirements, the majority of our sepsis patients clinically improved from ICU Day-1 to Day-3, raising the possibility that our data might not have adequately captured proteome changes in sepsis patients who clinically deteriorate, and highlights the need for future studies with subgroup analysis. Finally, we identified pathogens (culture positive sepsis) in only 33% of all sepsis patients; however, identification of pathogens by culture ranges from 28 to 89% of patients [115, 116].

## Conclusions

In conclusion, we present an exploratory study of high-throughput, targeted plasma proteomics from critically ill sepsis patients. Utilizing both machine learning and conventional statistics, we narrowed protein number for correlative investigations with clinical and laboratory parameters. Our exploratory data identified proteins that may serve as the targets for future hypothesis generating studies, including those investigating disease surveillance, organ dysfunction and outcome prediction. Furthermore, these data may be used to better understand the host response to both infection and interventions, including future studies investigating cellular and organ deconvolution, signalling pathways and drug repurposing.

## Supplementary Information

**Additional file 1: Table S1.** Protein function. **Table S2.** Median plasma expression of the top classifying proteins from sepsis patients for ICU Day-1 and ICU Day-3**. Table S3.** Correlations of dynamic change (Day 3 minus Day 1) between normalized plasma protein expression and sepsis patient parameters.

## Data Availability

The datasets generated and/or analysed during the current study are available from the corresponding author on reasonable request.

## Abbreviations

ICU: Intensive care unit
PEA: Proximity extension assay
P/F: PaO_2_/FiO_2_
MODS: Multiple Organ Dysfunction Scores
SOFA: Sequential Organ Failure Assessment Scores
IQR: Interquartile ranges
t-SNE: T-distributed stochastic nearest neighbour embedding
PRTN3: Proteinase 3
UPAR: Urokinase plasminogen activator surface receptor
GDF8: Growth differentiation factor 8
NTRK3: Neurotrophic receptor tyrosine kinase 3
WFDC2: WAP 4-disulphide core domain 2
CXCL13: Chemokine (C-X-C motif) ligand 13
IL6: Interleukin 6
IL10: Interleukin 10
CCL23: Chemokine (C–C motif) ligand 23
CCL20: Chemokine (C–C motif) ligand 20
CHI3L1: Chitinase 3 like 1
CPA2: Carboxypeptidase A2
DSG4: Desmoglein 4
CNTN5: Contactin 5
GCP5: Gamma-tubulin complex component 5
ITGAV: Integrin subunit alpha V
GALNT7: Polypeptide N-Acetylgalactosaminyltransferase 7
PLXDC1: Plexin domain containing 1
OPG: Osteoprotegerin
TPP1: Tripeptidyl peptidase 1
S100A11: S100 calcium binding protein A11
ELOA: Elongin A
FGR: Gardner-Rasheed feline sarcoma viral (v-fgr) oncogene homolog
SRP14: Signal recognition particle 14
TGFα1: Transforming growth factor α1
WFDC2: WAP four-disulfide core domain 2
INR: International normalized ratio
NTproBNP: NT-proB-type natriuretic peptide
ST2: Suppression of tumorigenicity 2 protein
ANGPT2: Angiopoietin-2
OPN: Osteopontin
ASGR1: Asialoglycoprotein receptor 1
TNFR1: Tumor necrosis factor receptor 1
TNFRSF10A: Tumor necrosis factor receptor superfamily member 10A
KIM1: Kidney injury molecule-1
