# Supplementary material for: Novel plasma protein biomarkers from critically ill sepsis patients
Source: Clin Proteomics. 2022 Dec 27;19:50. doi: 10.1186/s12014-022-09389-3 (PMC9792322; doi:10.1186/s12014-022-09389-3)
Supplement: Supplementary file 1 — Additional file 1: Table S1. Protein function. Table S2. Median plasma expression of the top classifying proteins from sepsis patients for ICU Day-1 and ICU Day-3. Table S3. Correlations of dynamic change (Day 3 minus Day 1) between normalized plasma protein expression and sepsis patient parameters. [file 12014_2022_9389_MOESM1_ESM.docx]

**Additional file 1: Table S1. Protein Function.**

| **Protein** | **UniProt ID** | **Function** |
| --- | --- | --- |
| **ANGPT2** | O15123 | Part of the angiopoietin family of growth factors. Interacts with TEK/Tie2 and AGPT2 to regulate endothelial cell apoptosis, migration and proliferation. Regulates angiogenesis. |
| **ASGR1** | P07306 | Cell surface transmembrane protein which mediates endocytosis and lysosomal degradation of glycoproteins. Appears to have roles in facilitating hepatic infection by various viruses. |
| **CCL20** | P78556 | Cytokine of the CC chemokine family. A ligand for the CCR7 chemokine receptor inducing chemotaxis of dendritic cells, T-cells and B-cells. Heavy role in mucosal immunity and inflammatory conditions such as cancer and autoimmune disease. Involved in intracellular calcium mobilization. |
| **CCL23** | P55773 | Cytokine of the CC chemokine family. Highly expressed in lung, liver, bone and placenta. Chemotactic for T-cells, monocytes and neutrophils. Inhibitory effect on hematopoiesis. |
| **CHI3L1** | P36222 | Secreted glycoprotein, Carbohydrate-binding lectin. Mediates the Th2 inflammatory response. Roles in inflammation, tissue remodeling, angiogenesis, Alzheimer’s disease, neurodegeneration, and cancer. |
| **CNTN5** | O94779 | Contactin protein known to mediate cell surface interactions. Largely characterized in neuronal development, and auditory pathology. |
| **CPA2** | P48052 | Carboxypeptidase protein along with CPA1, largely expressed in pancreatic tissue. A2 form acts on aromatic C-terminal residues. Exact function remains to be elucidated. |
| **CTSD** | P07339 | A ubiquitous lysosomal aspartyl protease, and member of the peptidase A1 family. Acts to degrade proteins and activate precursors in lysosomal compartments. Associated disease states include breast cancer, and neurologic disease. |
| **CXCL13** | O43927 | Member of the CXC chemokine family which bind to CXCR5. Chemotactic for B-cells but not T-cells, monocytes or neutrophils. |
| **DAG1** | Q14118 | Member of the dystrophin-associated glycoproteins, acting as a transmembrane link between the extracellular matrix and cytoskeleton. Widely distributed in muscle, epithelial and renal tissue. Known mutation in Duchenne muscular dystrophy. |
| **DSG4** | Q86SJ6 | Member of intercellular desmosome junction, involved in cellular adhesion and proliferation. |
| **ELOA** | Q14241 | Subunit of the transcription factor B (SIII) complex. General transcription elongation factor that increases RNA polymerase II transcription elongation via suppressing transient pausing of the polymerase. |
| **FGF21** | Q9NSA1 | Stimulates glucose uptake in differentiated adipocytes via GLUT1 expression. |
| **FGR** | PO9769 | Non-receptor tyrosine kinase that signals from the cell surface contributing to immune activation via regulating neutrophil, monocyte, macrophage and mast cell functions. Regulates cytoskeleton remodeling and cellular migration. Involved in a plethora of signaling cascades. |
| **GALNT7** | Q86SF2 | Type II transmembrane protein acting as a glycopeptide transferase involved in oligosaccharide biosynthesis. |
| **GCP5** | Q96RT8 | Component of the gamma tubulin complex which facilitates microtubule nucleation and centrosome assembly. |
| **GDF8** | A1C2F0 | A myokine that that is a member of the TGFβ family, produced by myocytes, which acts to inhibit muscle growth. Known roles in muscular dystrophy. |
| **GDF15** | Q99988 | Is abundantly expressed in placenta, and reproductive organs. However, is produced in many cell lineages under physiologic stress. Has roles in maintaining cellular and tissue homeostasis. Associated with inflammation, myocardial ischemia and malignancy. |
| **IL1RA** | B2KNI7 | Member of the interleukin 1 cytokine family. Binds to IL1 receptors but does not induce intracellular responses. Secreted by immune cells, epithelial cells, and adipocytes. Involved in cell proliferation, survival and differentiation. Regulates inflammatory responses. Has roles in autoimmune diseases and chronic inflammation. |
| **IL4RA** | P24391 | Type I transmembrane protein that binds IL4 and IL13 to regulate IgE production in B cells, differentiation of Th2 cells. Signals through the JAK/STAT pathway. |
| **IL6** | B5MCZ3 | Soluble mediator produced in response to infections and tissue injury. Has inhibitory effects on TNFα and IL1, while activating IL1RA and IL10. Involved in acute phase reactions, hematopoiesis, and immune reactions. Known to play a role in chronic inflammation and autoimmunity. |
| **IL10** | P22301 | Anti-inflammatory cytokine which largely signals through the JAK/STAT cellular pathway. Inhibits pro-inflammatory responses. Often acts to inhibit T-cell and macrophage function. Extensive documented roles in immune regulation. Numerous roles in human pathophysiology including allergy, infection, malignancy, inflammation and neurologic disease. |
| **ITGAV** | P06756 | Alpha-V integrin which acts as a receptor for a variety of extracellular ligands. Several roles in cellular signaling, migration, tumorigenesis. |
| **KIM1** | Q96D42 | Type I membrane protein with roles best characterized in renal disease, both acute and chronic. Also expressed in liver and spleen. |
| **NBN** | O60934 | Intracellular protein which works as a double-stranded DNA repair protein. Linked with roles in fertility, malignancy, immunoglobulin class switching. |
| **NPM1** | P06748 | Nucleolar ribonucleoprotein which promote ribosome biogenesis, protein chaperoning, histone formation, and shuttle small proteins to the nucleolus. Regulates cell proliferation and the tumor suppressor p53. |
| **NTRK3** | Q16288 | Receptor tyrosine kinase known to activate a variety of signaling pathways including MAPK and PIP3/AKT. Regulates cell survival and differentiation. |
| **NTproBNP** | P16860 | Cardiac hormone that is released upon increased cardiac wall tension to promote natriuresis, diuresis, vasorelaxation and inhibition of the renin and aldosterone system. Signals through the atrial natriuretic factor receptor. |
| **OPG** | O00300 | Decoy receptor for RANKL in the RANK/RANKL/OPG signaling pathway, inhibiting osteoclast differentiation and bone resorption. Binds TRAIL to induce cellular apoptosis. |
| **OPN** | Q3LGBO | Secreted acidic protein rich in negatively charged aspartic and glutamic acid. Substrate for several enzymes promoting inhibition of bone mineralization to regulate bone remodeling. Also expressed in several immune cells with several immunomodulating properties. Additional roles in cellular adhesion, wound healing and apoptosis. |
| **PLXDC1** | Q8IUK5 | Cell surface receptor for pigment epithelium derived factor (PEDF). Involved in endothelial cell morphogenesis and angiogenesis. |
| **PRTN3** | P24158 | Serine protease which degrades elastin, fibronectin, laminin, vitronectin, and collagen I/III/IV. Expressed in neutrophil granulocytes, regulates neutrophil migration, and involved in immune activation. |
| **PVR** | P15151 | Type I transmembrane glycoprotein in the immunoglobulin superfamily. Binds NK cell receptors CD96 and CD226 to form an immunological synapse between NK cells and target cells. Promotes secretion of lytic granules in activated NK cells. Additional roles in tumor invasion and migration. |
| **S100A11** | P31949 | Member of the S100 family of proteins which contain calcium binding motifs. Functions in cellular motility and invasion, and tubulin polymerization, as well as endo/exocytosis and apoptosis. Expressed in many tissues. |
| **SPINK1** | P00995 | Serine protease inhibitor with antitrypsin activity largely expressed in the pancreas to prevent premature activation of zymogens. |
| **SRP14** | P36108 | Cytoplasmic ribonucleoprotein involved in targeting secretory proteins to the endoplasmic reticulum. Complexes with SRP9 to constitute the arrest domain of SRP. |
| **ST2** | Q01638 | A member of the IL1R family, with membrane bound and soluble isoforms. Soluble form of ST2 binds IL33 to prevent IL33 from binding transmembrane ST2. Has roles in cardiac disease and cardio protection, and serves prognostic value after myocardial infarction. |
| **TGFα1** | A0A0X3P9T7 | Member of the EGF family and is a mitogenic peptide which binds kinase receptors to promote cell proliferation, differentiation and development. |
| **TIMP1** | P01033 | Metalloproteinase inhibitor and irreversibly inhibits them via binding to their catalytic zinc factor. Other roles in cell differentiation, migration and death via CD63 and ITGB1 signaling. |
| **TNFR1** | P19438 | One of the major cellular receptors for TNFα. Activates NF-kB, FADD, caspase signaling pathways to mediate apoptosis and regulate inflammation. |
| **TNFRSF10A** | O00220 | Cell surface receptor activated by TRAIL which promotes the cell death signal and induces apoptosis. Signaling occurs via recruitment of FADD and caspase-8 to the receptor complex. Promotes NF-kB signaling. |
| **TPP1** | O14773 | Serine protease located to the lysosome with peptidase action to generate tripeptides from proteolysis. High expression in bone marrow, placenta, lung, pineal gland and lymphocytes. |
| **UPAR** | Q03405 | Multidomain glycoprotein anchored by glycosylphosphatidylinositol to the cell membrane. Part of the plasminogen activation system to promote plasmin formation, regulating proteolysis. Involved in wound healing. |
| **VSIG4** | Q9Y279 | Related to the B7 family of immune regulatory proteins. Has roles in negative regulation of T cell proliferation, complement pathways and IL2 production. |
| **WFDC2** | Q14508 | Member of the WFDC family which has a WAP Signature motif of eight cysteines forming four disulfide bonds at the core of the protein to function as a protease inhibitor. Expressed in pulmonary epithelial cells and ovarian tissue. |

UniProt: the universal protein knowledgebase in 2021. Nucleic Acids Res 2021, 49(D1):D480-d489.

**Additional file 1: Table S2. Median plasma expression of the top classifying proteins from sepsis patients for ICU Day-1 and ICU Day-3.**

| Protein | Sepsis Day-1 | Sepsis Day-3 | Change* | P-Value |
| --- | --- | --- | --- | --- |
| IL1RA | 109.61 (83.37-142.19) | 73.40 (42.69-107.99) | **↓** | 0.0125 |
| IL6 | 169.24 (103.69-249.87) | 73.98 (29.30-151.58) | **↓** | 0.1205 |
| FGF21 | 498.68 (196.18-1735.47) | 203.50 (53.37-727.88) | **↓** | 0.0151 |
| OPG | 7.47 (5.29-10.82) | 6.01 (5.43-8.52) | **↓** | 0.3028 |
| GDF15 | 15.80 (12.49-30.66) | 16.20 (8.78-19.60) | **↑** | 0.0730 |
| PRTN3 | 22.72 (17.19-29.89) | 21.62 (15.90-27.30) | **↓** | 0.2524 |
| UPAR | 15.45 (11.52-30.35) | 13.75 (11.00-27.40) | **↓** | 0.3028 |
| CTSD | 6.75 (4.54-8.26) | 5.80 (4.08-6.74) | **↓** | 0.2293 |
| DAG1 | 1.34 (1.25-1.51) | 1.28 (1.10-1.37) | **↓** | 0.0256 |
| TPP1 | 32.29 (29.31-50.82) | 31.98 (29.28-34.93) | **↓** | 0.1354 |
| IL10 | 55.64 (32.67-98.75) | 22.12 (16.37-36.55) | **↓** | **0.0015** |
| CCL23 | 2299.48 (1512.89-3088.85) | 1519.10 (1176.10-2370.85) | **↓** | **0.0054** |
| CCL20 | 570.22 (401.45-819.50) | 289.45 (199.89-420.14) | **↓** | 0.0479 |
| SRP14 | 9.93 (7.00-19.83) | 8.75 (6.54-11.46) | **↓** | 0.0353 |
| CPA2 | 162.32 (79.99-329.63) | 262.91 (191.48-405.39) | **↑** | 0.0479 |
| GDF8 | 2.84 (2.54-3.48) | 2.11 (1.61-2.88) | **↓** | 0.1876 |
| NTRK3 | 67.96 (54.14-79.73) | 62.54 (49.32-70.67) | **↓** | 0.1205 |
| TGFα1 | 9.86 (7.20-16.83) | 8.32 (7.06-11.09) | **↓** | **0.0084** |
| S100A11 | 20.31 (16.91-22.85) | 16.15 (13.54-21.14) | **↓** | 0.0833 |
| WFDC2 | 851.42 (706.71-1441.42) | 985.66 (767.97-1208.01) | **↑** | 0.1876 |
| CXCL13 | 408.14 (300.90-493.60) | 446.75 (279.79-716.64) | **↑** | 0.2769 |
| ELOA | 1.02 (0.86-1.44) | 0.88 (0.66-1.03) | **↓** | 0.0181 |
| DSG4 | 2.80 (2.04-3.83) | 2.38 (1.80-3.52) | **↓** | 0.9341 |
| NBN | 4.89 (4.04-6.99) | 4.29 (3.24-5.24) | **↓** | 0.0637 |
| FGR | 10.66 (8.25-15.97) | 9.79 (6.95-12.84) | **↓** | 0.0833 |
| TIMP1 | 46.65 (29.72-86.18) | 40.17 (27.01-54.83) | **↓** | 0.0730 |
| IL4RA | 5.09 (4.62-12.57) | 6.56 (3.63-8.15) | **↑** | 0.0730 |
| KIM1 | 434.08 (271.80-784.47) | 590.88 (461.90-854.33) | **↑** | 0.0833 |
| OPN | 65.70 (29.22-93.90) | 70.09 (43.82-78.26) | **↑** | 0.8904 |
| CHI3L1 | 88.29 (67.51-115.82) | 51.95 (27.92-68.54) | **↓** | 0.0413 |
| ST2 | 78.90 (28.58-201.71) | 44.50 (16.73-72.03) | **↓** | **0.0054** |
| TNFR1 | 38.36 (30.51-104.97) | 49.37 (33.71-86.61) | **↑** | 0.3591 |
| TNFRSF10A | 9.79 (7.64-18.75) | 11.61 (7.28-14.22) | **↑** | 0.0151 |
| SPINK1 | 37.80 (10.31-173.10) | 75.56 (22.32-239.37) | **↑** | 0.7615 |
| VSIG4 | 70.37 (26.17-176.13) | 94.61 (47.58-203.81) | **↑** | **0.0034** |
| ANGPT2 | 10.85 (7.93-16.60) | 9.90 (7.78-16.06) | **↓** | 0.5614 |
| NTproBNP | 117.70 (33.68-1618.73) | 204.13 (66.21-817.22) | **↑** | 0.7615 |
| NPM1 | 32.94 (27.51-40.45) | 30.37 (22.76-38.50) | **↓** | 0.4543 |
| ASGR1 | 33.51 (21.40-45.83) | 32.71 (20.44-42.41) | **↓** | 0.3303 |
| CNTN5 | 9.53 (6.63-13.38) | 8.72 (6.28-10.84) | **↓** | **0.0034** |
| PVR | 337.71 (284.09-415.07) | 309.93 (285.85-389.24) | **↓** | 0.3591 |
| GCP5 | 6.32 (4.38-9.07) | 6.36 (4.92-8.15) | **↑** | 0.8904 |
| ITGAV | 18.71 (12.83-21.22) | 15.99 (11.29-17.31) | **↓** | **0.0012** |
| GALNT7 | 9.38 (7.73-11.34) | 9.52 (7.20-10.56) | **↑** | 0.1876 |
| PLXDC1 | 4.62 (4.29-5.52) | 4.71 (3.93-5.21) | **↑** | 0.1354 |

P<0.01 was considered significant to control for multiple comparisons.

* change represents the up-regulation (**↑**) or down-regulation (**↓**) from ICU Day-1 to ICU Day-3

**Additional file 1: Table S3. Correlations of dynamic change (Day 3 minus Day 1) between normalized plasma protein expression and sepsis patient parameters**

|  | **MODS** | **SOFA** | **P/F** | **Vaso** | **Ster** | **HgB** | **WBC** | **PMN** | **Lym** | **PLT** | **PTT** | **INR** | **Creat** | **Lact** |
| --- | --- | --- | --- | --- | --- | --- | --- | --- | --- | --- | --- | --- | --- | --- |
| **OPG** | .20 | -.10 | -.45 | -.14 | -.02 | .53 | .42 | **.74*** | -.24 | -.10 | **.76*** | **.66*** | -.11 | .06 |
| **PRTN3** | .22 | -.38 | -.48 | -.06 | .20 | **.55*** | **.67*** | **.85*** | **-.59*** | -.33 | **.67*** | .37 | -.15 | .02 |
| **UPAR** | -.35 | -.25 | .14 | -.49 | .20 | .02 | .03 | .05 | .35 | .41 | .30 | .33 | -.32 | .22 |
| **TPP1** | .27 | .12 | -.43 | -.03 | -.12 | .23 | .46 | .48 | -.26 | -.22 | **.72*** | .50 | .38 | .36 |
| **CCL23** | .33 | .35 | **-.59*** | -.12 | -.01 | -.14 | .10 | .16 | -.07 | -.14 | .12 | .05 | .09 | **.63*** |
| **CCL20** | -.40 | .17 | .24 | .11 | .01 | .33 | .04 | .11 | -.37 | .16 | .20 | .23 | .11 | **.59*** |
| **SRP14** | .22 | .33 | -.37 | .33 | -.20 | .35 | .09 | .28 | -.01 | -.24 | .11 | -.07 | .35 | -.38 |
| **CPA2** | -.36 | .53 | .40 | .23 | -.19 | -.28 | **-.70*** | **-.82*** | .50 | .31 | -.38 | -.09 | .14 | -.23 |
| **TGFα1** | .16 | .12 | -.29 | .35 | -.22 | .45 | .17 | .26 | -.39 | -.20 | **.66*** | **.74*** | -.09 | -.29 |
| **S100A11** | .24 | .20 | -.18 | .06 | -.01 | .31 | .40 | .47 | -.17 | -.25 | .14 | -.23 | .52 | -.22 |
| **WFDC2** | .31 | .21 | -.17 | .23 | -.13 | .27 | .23 | .23 | **-.73*** | -.28 | **.64*** | **.67*** | .21 | .22 |
| **CXCL13** | **.72*** | -.11 | **-.71*** | -.23 | .02 | -.05 | .43 | .50 | -.14 | -.45 | **.68*** | **.70*** | .07 | .07 |
| **ELOA** | .18 | .16 | -.28 | .17 | -.07 | .40 | .28 | .44 | .06 | -.08 | .10 | -.19 | **.56*** | -.15 |
| **FGR** | **.58*** | -.20 | -.52 | -.40 | .07 | .03 | **.55*** | **.66*** | -.28 | -.50 | **.65*** | .44 | -.10 | -.05 |
| **KIM1** | -.39 | .07 | .09 | -.33 | .22 | .33 | .31 | .37 | -.16 | .05 | **.56*** | .31 | -.10 | .08 |
| **OPN** | .02 | -.19 | -.43 | .32 | .04 | **.63*** | .33 | **.65*** | **-.73*** | -.27 | **.67*** | **.63*** | -.17 | .06 |
| **CHI3L1** | **.57*** | .16 | -.40 | .33 | -.14 | .30 | .40 | .49 | **-.79*** | **-.53*** | .48 | .26 | .09 | -.08 |
| **ST2** | .49 | .01 | -.48 | .17 | .12 | .11 | .22 | .37 | **-.69*** | -.47 | .32 | .32 | -.12 | .20 |
| **TNFR1** | -.25 | -.15 | .22 | .05 | -.17 | .34 | -.12 | -.02 | -.37 | .29 | .32 | .43 | -.51 | .33 |
| **TNFRSF10A** | -.32 | -.05 | .30 | .17 | .00 | .51 | .19 | .19 | **-.73*** | .04 | .36 | .30 | -.07 | .14 |
| **ANGPT2** | .20 | -.07 | -.14 | -.33 | -.08 | .23 | .20 | .28 | -.02 | .15 | **.67*** | **.81*** | -.11 | .21 |
| **NTproBNP** | .44 | -.29 | **-.59*** | -.22 | .16 | -.18 | **.57*** | **.70*** | -.08 | **-.70*** | .28 | .16 | -.17 | .06 |
| **ASGR1** | .05 | .09 | -.25 | -.05 | .08 | .32 | .15 | .22 | -.42 | -.03 | **.65*** | **.55*** | -.15 | .03 |
| **GCP5** | .03 | .07 | .19 | -.30 | -.42 | -.07 | -.06 | -.16 | **.72*** | .41 | -.08 | .03 | .15 | -.09 |
| **ITGAV** | -.37 | .05 | **.72*** | -.22 | -.07 | .13 | -.11 | -.23 | -.06 | **.54*** | -.38 | **-.54*** | .30 | .28 |
| **PLXDC1** | **-.58*** | -.18 | .06 | .04 | -.05 | .19 | .06 | .05 | .15 | .09 | .24 | .17 | -.36 | .00 |

Red represents the up-regulation and Blue indicates down-regulation from ICU Day-1 to ICU Day-3. Abbreviations: MODS – Multiple Organ Dysfunction Score; SOFA - Sequential Organ Failure Assessment; P/F - arterial partial pressure of oxygen divided by the fraction of inspired oxygen concentration; Vaso – vasopressors; Ster – steroid; HgB – hemoglobin concentration; WBC – white blood cell count; PMN – neutrophil count; Lym – lymphocyte count; PLT – platelet count; PTT - partial thromboplastin time; INR – international normalized ratio; Creat – creatinine concentration; Lact – lactate. Mechanical ventilation had no change from Day 1 to Day 3, so was excluded from the table. * p<0.05.
